# Supplementary figures and images for: Temporal dynamics of gene expression in heat-stressed Caenorhabditis elegans
Source: PLoS One. 2017 Dec 11;12(12):e0189445. doi: 10.1371/journal.pone.0189445 (PMC5724892; doi:10.1371/journal.pone.0189445)

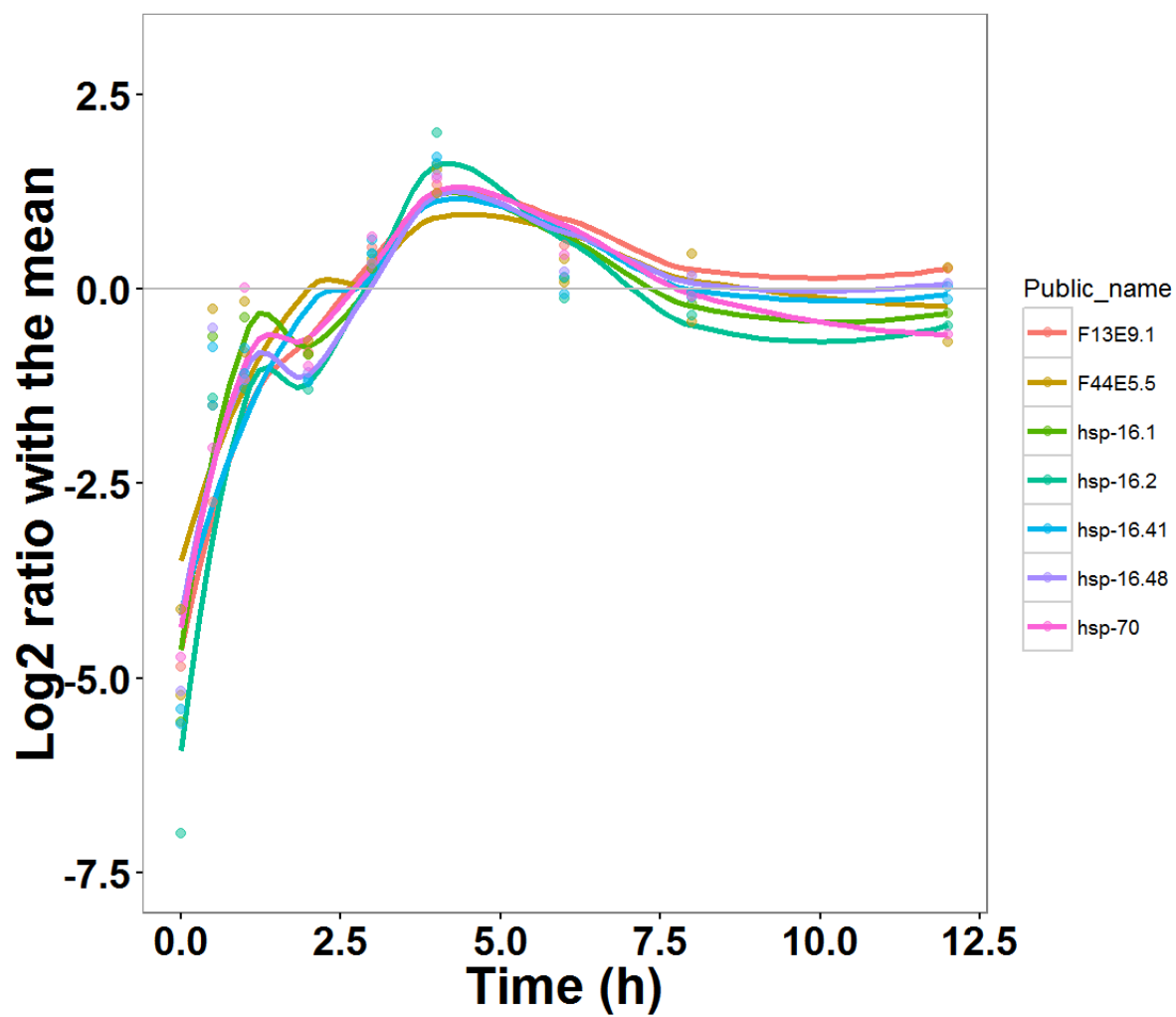

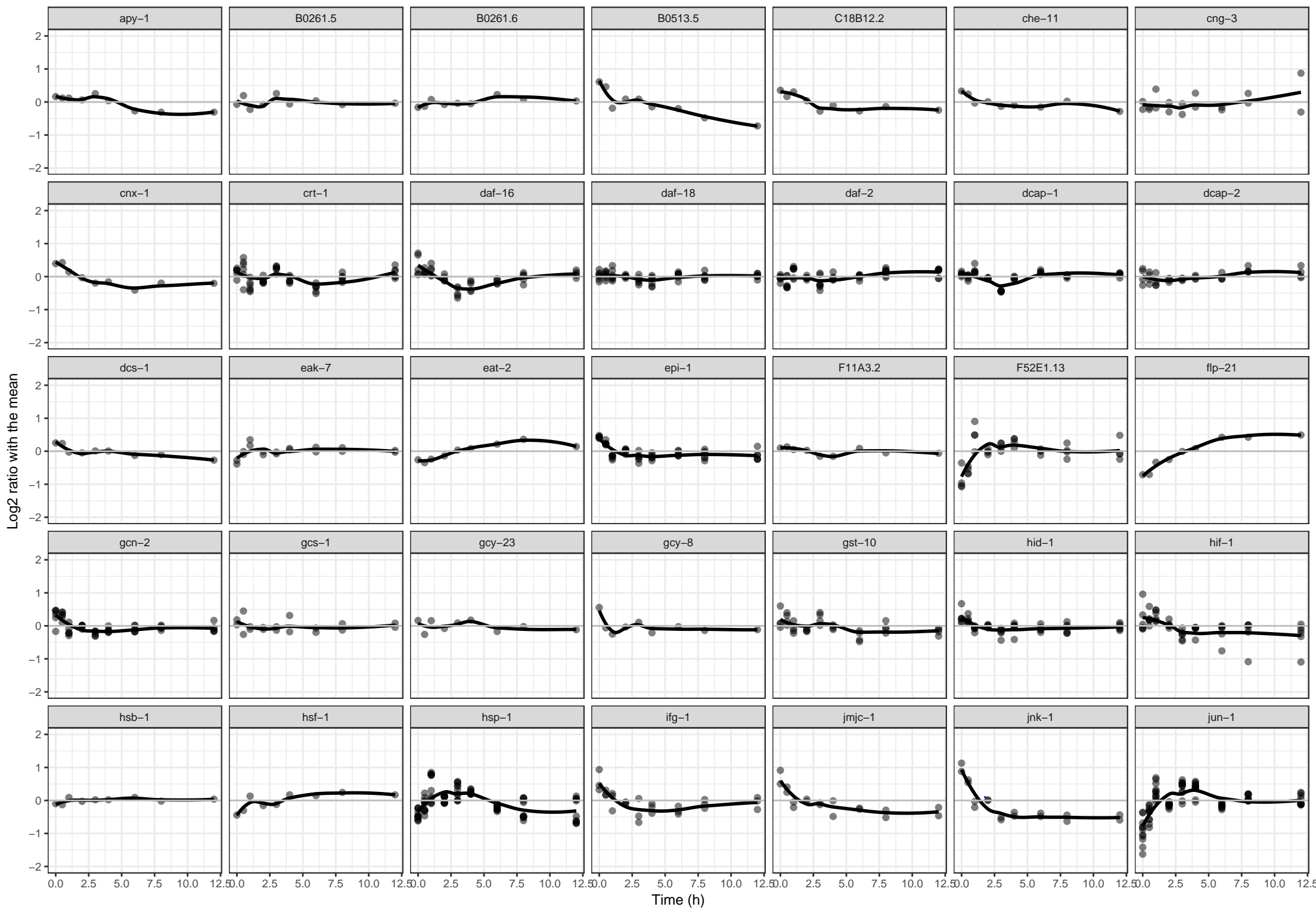

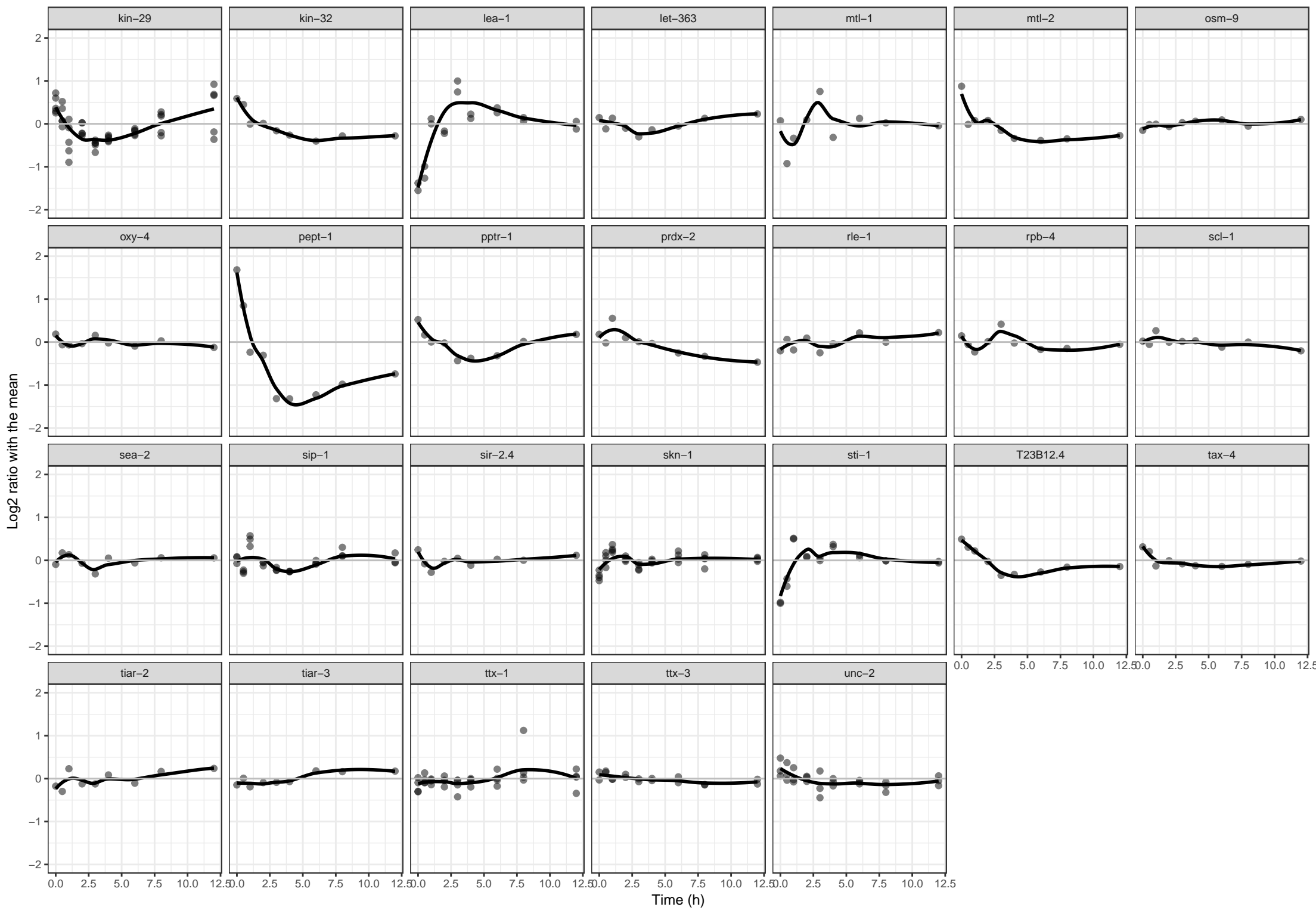

Supplement: S1 Fig — Genes were selected based on the information provided by the Gene Ontology database for the GO term ‘response to heat stress’ (GO:0009408, WormBase version 257). Expression levels of individual genes are presented as the Log2 ratio with the mean. (PDF) [file pone.0189445.s001.pdf]

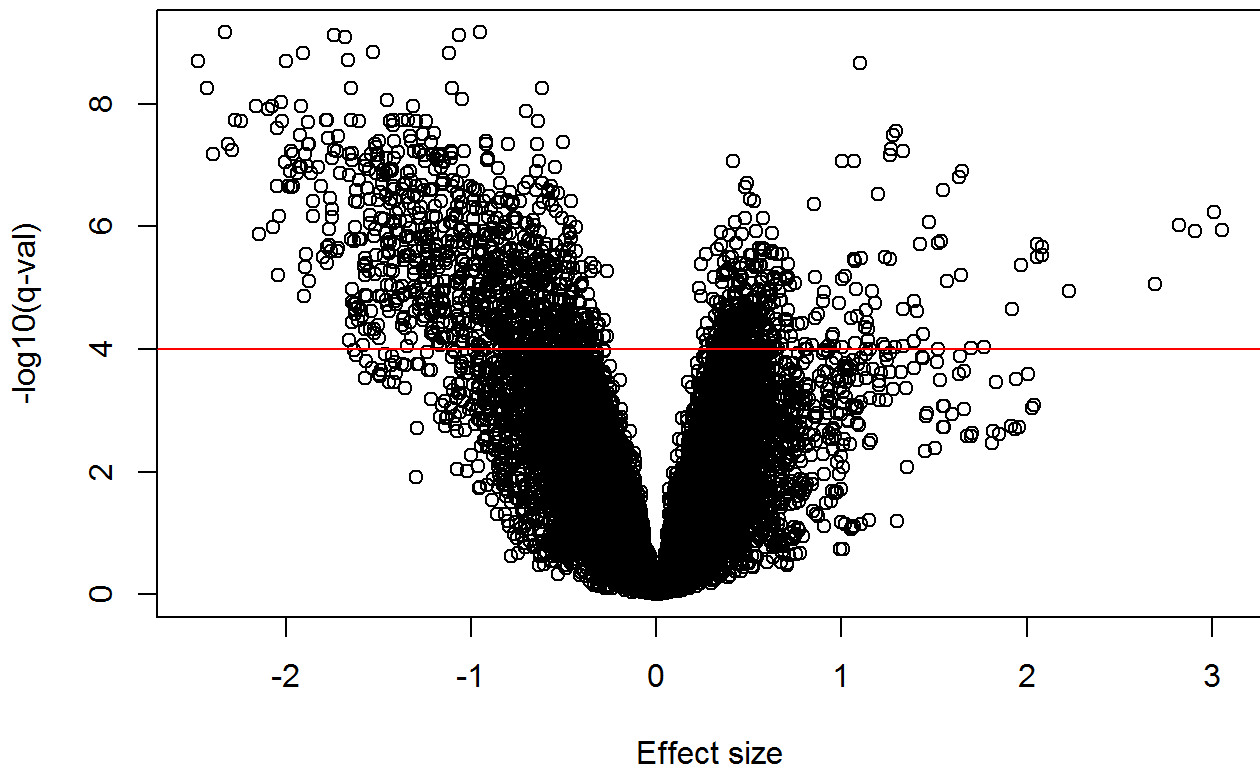

Supplement: S2 Fig — P-values were inferred from a linear model comparing the two groups, and corrected for multiple testing by the Benjamini-Hochberg method. The red line indicates the selected significance level resulting in the selection of 262 upregulated (positive effect size) and 667 downregulated (negative effect size) genes. (PDF) [file pone.0189445.s002.pdf]

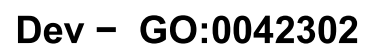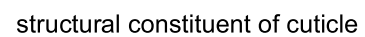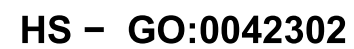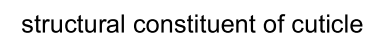

Supplement: S3 Fig — Developmental gene expression data was obtained from Snoek et al. 2014. Genes were selected based on the information provided by the Gene Ontology database for the GO term ‘structural constituents of the cuticle’ (GO:0042302, WormBase version 257). (PDF) [file pone.0189445.s003.pdf]
